# Antimicrobial effect of silver nanoparticles on polypropylene and acrylic resin denture bases

**DOI:** 10.4317/jced.59766

**Published:** 2023-01-01

**Authors:** Márcio-Katsuyoshi Mukai, Carolina-Mayumi Iegami, Silvana Cai, Roberto-Chaib Stegun, Alessandra-Pucci-Mantelli Galhardo, Bruno Costa

**Affiliations:** 1 School of Dentistry, University of São Paulo, São Paulo, São Paulo, Brazil; 2 Biomedical Sciences Institute, University of São Paulo, São Paulo São Paulo, Brazil; 3 Medical School, Anhanguera Uniderp University, Campo Grande, Mato Grosso do Sul, Brazil

## Abstract

**Background:**

The investigation of the antimicrobial action of Ag solutions in different surfaces has been performed, however, there is little data on the direct addition of those particles to the polypropylene denture base. The aim of this study was to evaluate the antimicrobial effect of silver nanoparticles in *Candida albicans* and *Streptococcus mutans* colonization in acrylic resin and polypropylene denture bases.

**Material and Methods:**

Twenty test bodies (10×13×3 mm) were fabricated with (heat-polymerized) poly methyl methacrylate acrylic resin (group 1), (heat-polymerized) poly methyl methacrylate acrylic resin with silver nanoparticles (group 2), polypropylene (group 3) and polypropylene with silver nanoparticles (group 4). *Candida albicans* and *Streptococcus mutans* colonization were performed. A colony count was performed in Colony Forming Unit (CFU/ml) and registered to calculate the arithmetical mean of the duplicate values of each sample. Data was analyzed by Lilliefors test and since samples did not present a normal distribution, Kruskal-Wallis for variance analysis with the value of significance set at *p*<0.05.

**Results:**

For *Candida albicans* colonies, when comparing nano-Ag in polypropylene, there were statistical difference between groups 3 and 4. For the same type of growth in heat-polymerized PMMA resin with and without nano-Ag, no statistical differences were found. The presence of nanoparticles did not affect *Streptococcus mutans* growth in the heat-polymerized PMMA resin. Value differences of *S. mutans* were not significant for comparisons between groups 1-2 and 3-4.

**Conclusions:**

Silver nanoparticles presented antifungal activity against C. albicans in polypropylene surfaces, but not in the heat-polymerized PMMA resin. Silver nanoparticles did not present antibacterial activity against *S. mutans* in any of the analyzed surfaces.

** Key words:**Removable Partial Denture, Candida, Streptococcus mutans.

## Introduction

Aesthetics is one of the main concerns in dentistry. Dental materials have been developed in order to mimic the natural structures, however, in removable partial dentures (RDPs), the exposure of metal clasps often is the cause of frustration among RDP wearers (1). In response, manufacturers have introduced several materials, for example thermoplastic resins, as an alternative to the metal and the heat-polymerized polymethylmethacrylate (PMMA) resin. These materials have been tested for numerous conditions, such as physical properties, color stability and cytotoxicity, in order to create the best material that is both aesthetical and functional.

Fungal and bacterial adherence to the denture base is frequently found both in PMMA and thermoplastic resins used in RDPs. Its wearers are often afflicted with denture stomatitis, a condition characterized as an inflammation of the mucosa covered by the removable prosthesis (2-4). The etiology appears to be multifactorial, including a pathogenic response to **Candida* albicans* (2). Problems such as poor hygiene, uninterrupted use and ill-fitting dentures might increase the progress of denture stomatitis. Although these lesions can be prevented with denture cleansing and antifungal agents, in some cases the funguses are resistant and or denture cleaning is not effective.

Antimicrobial agents have been added to the denture base in order to prevent the growth of microorganisms, such as drugs and inorganic compounds. Silver (Ag) has been recognized as an excellent antimicrobial agent, either in the form of ions or nanoparticles (5-7). The investigation of the antimicrobial action of Ag solutions in different surfaces has been performed (8-10), however, there is little data on the direct addition of those particles to the polypropylene denture base. 

The aim of this *in vitro* study was to evaluate antimicrobial activity in heat-polymerized and polypropylene denture bases combined to Ag nanoparticles (nano-Ag) against *S. mutans* and *C. albicans*.

## Material and Methods

Twenty specimens were fabricated for each group: Group 1 – heat-polymerized PMMA resin without nano-Ag (RTC) (Acrílico Termo-Polimerizante; Artigos Odontológicos Clássico, São Paulo, Brazil), Group 2 – heat-polymerized PMMA resin with nano-Ag (RTN), Group 3 – polypropylene (PPC) (Rocalflex; Essence Dental Importação e Exportação Ltda, São Paulo, Brazil) and Group 4 – polypropylene with nano-Ag (PPN).

For groups 1 and 2, 4 grams of polymer were added to 1.9 ml of monomer and the blend was poured in to a U-shaped aluminum mold of 10×130×3 mm. The mold was placed in a metal flask and went through the curing cycle recommended by the manufacturer (70ºC for 30 minutes, 100ºC for 1.5 h). Subsequently, the U-shaped model was cut into strips of 10×10×3 mm. In order to fabricate specimens for Group 2, a solution 30 ml of nano-Ag (80 parts per million – ppm) (Prata Nanoparticulada; Khemia Equipamentos Tecnológicos de Efluentes Ltda., São Paulo, Brazil) was manually mixed to 100g of heat-polymerized PMMA resin (Acrílico Termo-Polimerizante; Artigos Odontológicos Clássico, São Paulo, Brazil). After drying in an oven (Olidef, Ribeirão Preto, Brazil) at 37ºC for 48 hours, the mixture was manually homogenized and cured in the U-shaped mold as previously described.

For groups 3 and 4, polypropylene spheres (Rocalflex; Essence Dental Importação e Exportação Ltda, São Paulo, Brazil) were placed in an aluminum tube and heated in an oven at 270ºC for 14 minutes. The material was injected in to a stainless-steel mold of 10×130×3 mm. The sample was cut in to strips of 10×10×3 mm.

For group 4, nano-Ag was diluted in a specific pigment and applied to the polypropylene spheres before placement in the aluminum tube.

The specimens were individually packed and sterilized with a dose of 25kGy of gamma irradiation at the Centro de Tecnologia das Radiações – CTR IPEN/CNEN/SP.

Test-tubes containing 1 ml of Tryptic Soy Broth (TBS) culture medium for *Candida albicans* (ATCC 10,231) and *Streptococcus mutans* bacteria were used to individually inoculate the microoganisms at 37ºC for 24 hours. After this period, the inoculated culture medium was adjusted to 1 Mc Farland standard for *Candida albicans* and *Streptococcus mutans*.

Specimens from all the groups were randomly distributed in a cell culture dish with 1 ml of *Candida albicans* culture media. The same procedure was executed for *Streptococcus mutans* culture media. After inoculation, the specimen dishes were incubated for 1.5 hours under vibration at intensity 1 (Vortex Genie 2, Scientific Industries, Inc., Bohemia, New York, USA) and washed with saline solution in 2 cycles of 1 minute each. The samples were individually transferred to orifices of a new cellular culture dish with 24 holes. The dish contained 2ml of TBS and was incubated at 37ºC for 48 hours, in order to mature the biofilm. Specimens were removed and individually transferred to ependorf tubes filled with 1 ml of sterile saline solution and 3 pearls of glass. The tubes were agitated for 1 minute in a vibrator with the purpose of detaching any cell adhered to the test body.

Serial dilutions were performed from the resultant solution (10-3,10-4,10-5,10-6), followed by seeding with 3 drops of 10 µl for each concentration in the Petri dish containing the Tryptic Soy Agar (TSA). The dishes were incubated at 37ºC for 24 hours.

A colony count was performed in Colony Forming Unit (CFU/ml) and registered to calculate the arithmetical mean of the duplicate values of each sample.

Data was analyzed by Lilliefors test and since samples did not present a normal distribution, Kruskal-Wallis for variance analysis with the value of significance set at *p*<0.05.

## Results

**Figure 1:**
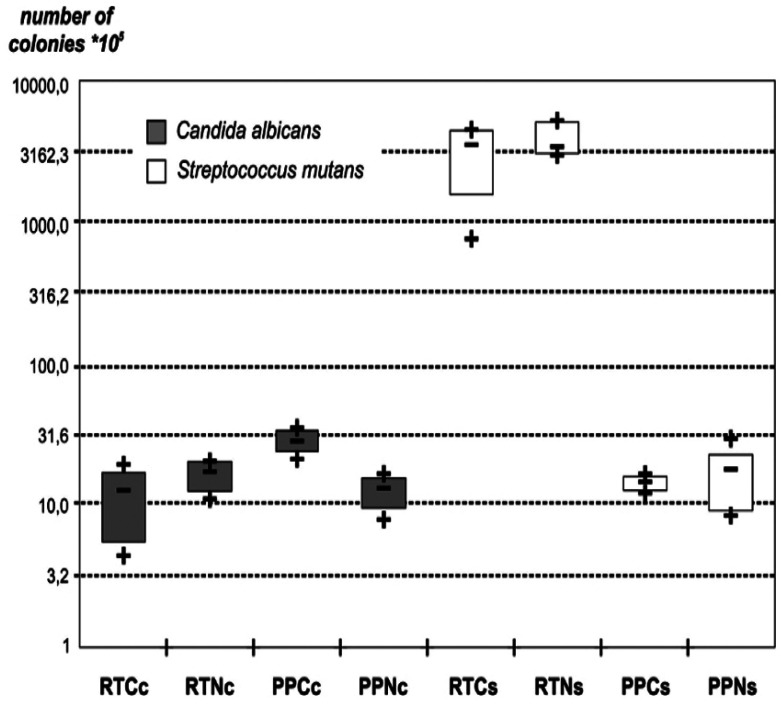
Arithmetical mean of *Candida albicans* colonies count and Streptococcus mutans colonies count, converted in log.

The arithmetical mean of *Candida albicans* colonies and *Streptococcus mutans* colonies are displayed in Figure 1.

**Figure 2:**
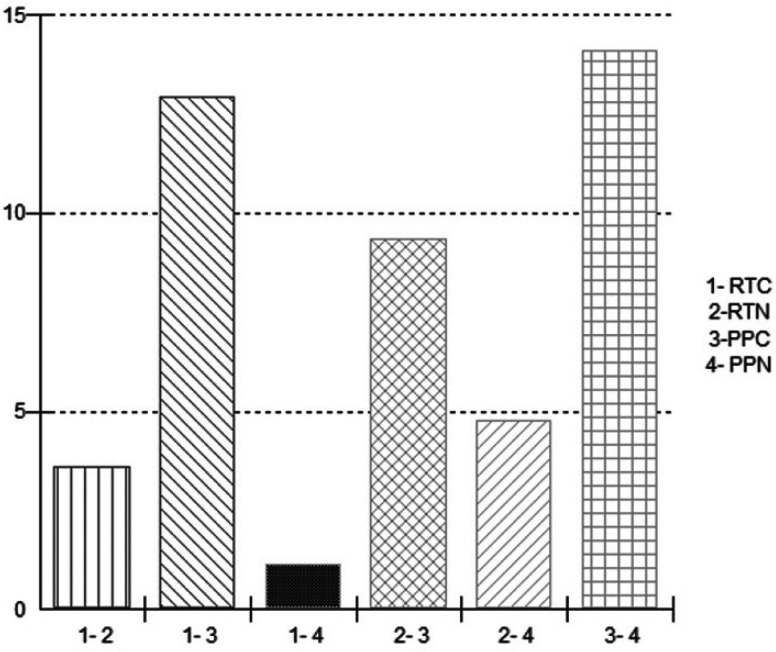
Comparison of the effect of nano-Ag in *Candida albicans*.

For *Candida albicans* colonies, the analysis shows that when comparing nano-Ag in polypropylene, there is statistical difference between groups 3 and 4 (Fig. 2). For the same type of growth in heat-polymerized PMMA resin with and without nano-Ag, no statistical differences were found.

**Figure 3:**
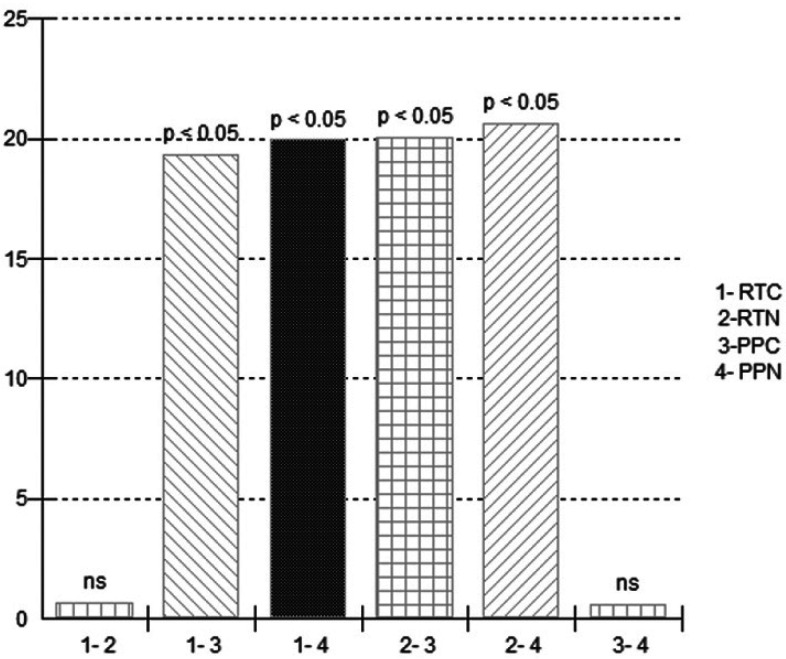
Comparison of the effect of nano-Ag in *Strepcoccus mutans*.

According to the results, the presence of nanoparticles did not affect *Streptcoccus mutans* growth in the heat-polymerized PMMA resin. Value differences of *S. mutans* were not significant for comparisons between groups 1-2 and 3-4 (Fig. 3).

Although there was no statistical difference between heat-polymerized PMMA resin with and without nano-Ag for both types of colonization, surface adherence of *Streptococcus mutans* was statistically lower to the polypropylene than to the heat-polymerized PMMA resin.

## Discussion

Silver has been recognized as an excellent antimicrobial agent in the form of ions, as well as in nanoparticles (5-7) and it has been applied in the medical field for such characteristic (11).

Some studies (8,12) have investigated the antimicrobial action of Ag nanoparticles. However, there is little data on the direct incorporation of those to denture bases, the way it was performed in this study. The colloidal solution was dehydrated, in order to avoid gathering of the particles within the polymer, improving its distribution. The resulting powder, with 80 ppm of nano-Ag did not present color alterations, unlike previous studies (13).

The antimicrobial activity of Ag nanoparticles in denture bases was analyzed with viable colony count. The analysis of the results of *C. albicans* colonization showed that the addition of Ag nanoparticles is not statistically significant in relation to heat polymerized resins. While some studies show the effectiveness of nano-Ag on heat polymerized resins, similar results were presented by other authors (13,14).

The hydrophobicity of the polymerical surfaces is an important factor in the process of fungus adhesion and biofilm formation (15). The addition of Ag nanoparticles might have altered the physical-chemical characteristics of the PMMA/Ag nanocomposite, interfering with it.

The antimicrobial effect of nano-Ag is dependent on its size: the smaller the particle size, the more effective is the binding to the bacteria. Smaller particles, preferably with larger surface area will be more bactericidal than larger particles (7,15,16). The incorporation of Ag microparticles makes the composite resin more hydrophobic, which is exacerbated with the enhancement of the concentration of Ag particles (18). Another factor that might have influenced the behavior of the material is the difficult homogenization of the nano-Ag colloid in the resin mass during the laboratorial process. It might have prevented the nanoparticles dispersion, generating more aggregation and possibly leading the combined particles to behave as a big single mass, instead of various smaller units with high reactivity (19).

The results might also have been affected by the structure of the polymeric matrix. The acrylic resin monomer presents a cross-linked agent (ethylene glycol dimethacrylate). Therefore, it is likely that due to the polymeric structure formed in the samples, silver ions or Ag nanoparticles were retained in the reticulated structure of the polymer and the liberation of those was limited.

The mechanism by which the nanoparticles interact with the bacteria is not very well-known, however studies suggest that the attachment of nanoparticles to the surface of the cell membrane causes a modification in its permeability (7,20). Additionally, it has been demonstrated through increase of the values of intracellular glucose and trehalose that nano-Ag can cause a depolarization of the fungal membrane and its breaking (21).

*Candida albicans* is known to colonize various surfaces, but the adhesion to each niche is different (22). The polypropylene shows efficiency of the material towards the acrylic resin in the matter of antimicrobial activity, with (group 4) or without Ag nanoparticles (group 3). The adherence of *Candida albicans* and *Streptococcus mutans*, when comparing both materials, was smaller in the polypropylene than in the acrylic resin. Factors such as difference of hydrophobicity and surface porosity might have influenced these results.

## Conclusions

Within the limitations of this *in vitro* study, it can be concluded that the nano-Ag solution presented antifungal activity against *C. albicans* in polypropylene surfaces, but not in the heat-polymerized PMMA resin. Ag nanoparticles solution did not present antibacterial activity against *S. mutans* in any of the analyzed surfaces.
